# Predicting Outcomes in Patients With Diffuse Large B-Cell Lymphoma Treated With Standard of Care

**DOI:** 10.1177/1176935119835538

**Published:** 2019-03-15

**Authors:** Aaron Galaznik, Christian Reich, Greg Klebanov, Yuriy Khoma, Eldar Allakhverdiiev, Greg Hather, Yaping Shou

**Affiliations:** 1 Millennium Pharmaceuticals, Inc., a wholly owned subsidiary of Takeda Pharmaceutical Company Limited, Cambridge, MA, USA; 2 IMS Health, Danbury, CT, USA; 3 ‎Odysseus Data Services, Inc., Cambridge, MA, USA; 4 Lviv Polytechnic National University, Lviv, Ukraine

**Keywords:** algorithm, DLBCL, health outcomes, observation period, predictive model, predictor, regression, targeted drug development, therapy supplemental

## Abstract

In diffuse large B-cell lymphoma (DLBCL), predictive modeling may contribute to targeted drug development by enrichment of the study populations enrolled in clinical trials of DLBCL investigational drugs to include patients with lower likelihood of responding to standard of care. In clinical practice, predictive modeling has the potential to optimize therapy choices in DLBCL. The objectives of this study were to create a model for predicting health outcomes in patients with DLBCL treated with standard of care and determine informative predictors of health outcomes for patients with DLBCL. This was a retrospective observational study using data extracted from the IMS Health Database between September 2007 and April 2015. Patients were ⩾18 years of age with a DLBCL diagnosis. The index date was the date of the first DLBCL diagnosis. Patients were followed until outcome occurrence, defined as progression to a later line of therapy after ⩾60 days from the end of a previous therapy or stem cell transplantation. Patients were categorized into three cohorts depending on the post-index observation period: ⩽1 year, ⩽3 years, or ⩽5 years. Lasso logistic regression (LASSO), Naive Bayes, gradient-boosting machine (GBM), random forest (RF), and neural network models were performed for each cohort. The best-performing algorithms were predictive models based on GBM and observation periods ⩽1 and ⩽3 years after index date. Informative predictors included myocardial imaging, DLBCL stage IV, bronchiolar and renal disease, a chemotherapy regimen, and exposure to diphenhydramine and vasoprotectives on or before the first DLBCL diagnosis. These predictive models may be applied to targeted drug development and have the potential to optimize therapy choices in DLBCL. They were generated efficiently using a large number of independent variables readily available in standard insurance claims or electronic health record data systems.

## Background

Non-Hodgkin lymphoma (NHL) is a heterogeneous family of lymphoid malignancies, which typically develop in lymph nodes but may occur in almost any tissue. In the United States, between 2010 and 2014, the incidence of NHL was 23.7 per 100 000 individuals in men and 16.0 per 100 000 individuals in women.^1^

Approximately 10% to 15% of NHL is derived from T cells or natural killer cells, but most cases (85%-90%) are of B-cell origin. In the United States, diffuse large B-cell lymphomas (DLBCL) account for 30% to 40% of all NHL cases diagnosed each year.^2^ Between 2002 and 2011, there were approximately 56 521 new cases of DLBCL in the United States,^3^ mostly in older adults, as median age at diagnosis is 65 years.^4^

Standard first-line therapy for DLBCL is chemotherapy, usually with rituximab, cyclophosphamide, doxorubicin, vincristine, and prednisone (R-CHOP).^5^ This regimen is beneficial in many patients, but 10% to 20% of patients with limited stage disease at presentation and 30% to 50% of patients with advanced-stage disease experience relapse after first-line therapy,^6^ and 10% to 15% of patients fail to achieve complete response and are considered to have primary refractory disease.^7^ The clinical approach to relapsed/refractory DLBCL is high-dose chemotherapy without or with autologous stem cell transplantation; however, these regimens can only achieve a cure in 40% to 50% of patients.^7^ Diffuse large B-cell lymphoma treatment, beyond first-line therapy, is costly. In the United States, annual expenditures for non-relapsers to first-line therapy are estimated at US$25 004, rising to US$174 928 and US$301 426 in relapse patients treated without and with autologous stem cell transplantation, respectively.^8^

Management of DLBCL remains a challenge, and advances and further evaluation of investigational treatment options are required to improve patient outcomes. Increasingly, modeling is used to predict outcomes for individual patients in oncology.^9^ Predictive modeling is a process that uses data mining and probability to forecast patient responses to treatment. Each model comprise a number of predictors, which are variables that are likely to influence response or resistance to treatment. Once data have been collected for relevant predictors, a statistical model is formulated. In DLBCL, predictive modeling can contribute to targeted drug development by supporting recruitment decisions in clinical trials and has the potential to optimize therapy choices in clinical practice.

In the current treatment environment, clinical trials of investigational drugs in DLBCL must focus on patients with lower likelihood of responding to standard of care. As such, the design of clinical trials in DLBCL may be improved by enrichment of the study population, defined as selecting a study population in which detection of a drug effect (if one exists) is more likely than it would be in an unselected population.^10^ Enrichment of a DLBCL study population may be achieved using a predictive model for response rate to standard of care, whereby a population of non-responders is identified and randomized to either the new drug or the original one.

In clinical practice, a predictive model can be used to identify patients with DLBCL that have an increased probability of response to a specific treatment.^9,10^ Patient stratification based on a combination of selective variables can facilitate optimal therapy choices in DLBCL and improve the success rate of treatments. Furthermore, this approach could decrease the burden of DLBCL disease and reduce DLBCL health care costs by allowing comprehensive risk assessments and improved efficiencies in the delivery of care to DLBCL patients.

Although DLBCL has prognostic indicators, such as the International Prognostic Index (IPI)^11^ and known biomarkers associated with disease responsiveness, to our knowledge, there are no predictive models of treatment response rates in DLBCL. Furthermore, outside of clinical trial or registry settings, these prognostic indicators and biomarkers are usually not readily available in secondary data sources, such as insurance claims or electronic health records. The objectives of this study were to (1) create a model for predicting health outcomes in patients with DLBCL treated with standard-of-care therapy and (2) base the model on variables readily available in standard insurance claims or electronic health record data systems.

## Methods

### Data sources

This retrospective observational study used data extracted from the IQVIA Real-World Data Adjudicated Claims (PharMetrics Plus) database between September 2007 and April 2015.^12,13^

### Study design

Patients with DLBCL were eligible for this study. Inclusion criteria were as follows: (1) ⩾18 years of age; (2) ⩾one claim with a DLBCL diagnosis code in any position on an inpatient or outpatient record (Table 1); and (3) ⩾6 months of enrollment before the index date and ⩽1 year, ⩽3 years, or ⩽5 years of enrollment after the index date, depending on the length of the prediction window. The ⩾6 months pre-index enrollment requirement was to provide adequate characterization of baseline characteristics and identify potential oncology treatments before the index date (ie to reduce misclassification of incident newly diagnosed patients).

**Table 1.** Diagnosis codes.

Condition	ICD-9 codes
DLBCL	200.7x 202.0x
Other primary cancer and metastatic disease	140.xx-172.xx, 174.xx-176.xx, 179.xx-189.x, 190.x-199.xx, 201.xx, 203.xx-204.xx, 206.xx-208.xx, 209.0x-209.3x, 235.xx-237.xx, 238.0-238.6, 238.8-238.9

Abbreviations: DLBCL, diffuse large B-cell lymphoma; ICD-9, International Classification of Diseases, Ninth Revision.

Exclusion criteria were as follows: (1) diagnosis of DLBCL during the 6 months before the index date; (2) ⩾one claim with a diagnosis code for other primary cancer in any position on an inpatient or outpatient record (nodular lymphoma [ICD 202.0] if it first occurred within 30 days of a large cell lymphoma code was not excluded, in case of early misdiagnosis) (Table 1); or (3) ⩾one claim with a diagnosis code for secondary cancer (metastatic disease) in any position on an inpatient or outpatient record (Table 1).

The index date was the date of the first DLBCL diagnosis. Patients were followed until outcome occurrence and categorized into three cohorts depending on the post-index observation period: ⩽1 year, ⩽ 3 years, or ⩽ 5 years.

### Data collection

Outcomes assessment was binary, with patients being categorized as either disease progression or non-progression after first-line treatment. Due to a lack of granular treatment response data in insurance claims data, a proxy was used: initiation of a later line of therapy after ⩾60 days from the end of a previous therapy or stem cell transplantation, as identified by ICD-9 procedure, Healthcare Common Procedure Coding System (HCPCS), or Current Procedural Terminology (CPT) codes (Table 2).

**Table 2.** Treatment codes.

Description	Codes
Drugs	
	HCPCS
Bendamustine	J9033, C9243
Carboplatin	J9045
Cisplatin	J9060, J9062
Cyclophosphamide	J8530, J9070, J9080, J9090-J9097
Cytarabine	J9100, J9110, J9098 (liposomal)
Doxorubicin	J9000; pegylated liposomal: J9001, J9002, Q2048, Q2049, Q2050
Etoposide	J8560, J9181, J9182
Gemcitabine	J9201
Ifosfamide	J9208
Lenalidomide	None
Methotrexate	J8610, J9250, J9260
Mitoxantrone	J9293
Oxaliplatin	J9263
Procarbazine	S0182
Rituximab	J9310
Vincristine	J9370, J9371 (liposomal), J9375, J9380
Stem cell transplant	38240, 38241, 38243, S2142 41.00, 41.01, 41.02, 41.03, 41.04, 41.05, 41.06, 41.07, 41.08, 41.09
Transfusions (RBC, platelet, unknown)	36430, 36455, 86950 99.01, 99.02, 99.03, 99.04, 99.05, 99.06, 99.07
G(M)-CSF, n (%)	J1440, J1441, J1442, J1446, J2505, J2820, Q5101
Erythropoiesis-stimulating agents	J0881, J0885, Q4081
Stem cell transplantation	
	ICD-9 procedure
Autologous hematopoietic stem cell transplant without purging	41.04
Autologous hematopoietic stem cell transplant with purging	41.07
Bone marrow transplant, not otherwise specified	41.00
Autologous bone marrow transplant without purging	41.01
Allogeneic bone marrow transplant with purging	41.02
Allogeneic bone marrow transplant without purging	41.03
Allogeneic hematopoietic stem cell transplant without purging	41.05
Cord blood stem cell transplant	41.06
Allogeneic hematopoietic stem cell transplant with purging	41.08
Autologous bone marrow transplant with purging	41.09
	CPT
Hematopoietic progenitor cell (HPC); allogeneic transplantation per donor	38240
Transplantation of patient’s bone marrow or blood-derived stem cells	38241
Transplantation of donor bone marrow or blood-derived stem cells	38243
	HCPSC
Cord blood-derived stem cell transplantation, allogeneic	S2142

Abbreviations: CPT, current procedural terminology; CSF, colony-stimulating factor; HCPSC, Healthcare Common Procedure Coding System; ICD-9, International Classification of Diseases, Ninth Revision; RBC, red blood cell.

Mortality data are not available in the IQVIA PharmetricsPlus database. To avoid confounding, potentially deceased patients (defined as patients with an enrollment period that ended without an outcome before the end of the post-index observation period) were excluded from data analysis.

### Statistical analysis

Statistical analyses were conducted using the OHDSI R packages patient-level prediction, Cyclops, Cohort Method, DatabaseConnector, SqlRender, FeatureExtraction, and others.^14–19^ Some of the analyses were performed using the R packages BigKnn and xgboost, as well as the python sci-kit learn library tools.^20–22^

Select descriptive characteristics were assessed for each cohort based on availability of data; continuous measures were summarized as means and standard deviations, whereas categorical measures were summarized as counts and percentages (Tables 3 to 5). Supporting medications included erythropoiesis agents, granulocyte colony-stimulating factor (G-CSF) or granulocyte-macrophage colony-stimulating factor (GM-CSF), and blood transfusions. Pain medications and antifungals were not considered as predictors because of their potential use for other conditions.

**Table 3.** Descriptive statistics for DLBCL Cohort 1, observation period ≤1year after index date.

Variable	All subjects (N = 4501)	Outcome (N = 1646)	No outcome (N = 2855)
Age (mean, SD)	56.33 (13.74)	57.12 (13.74)	55.88 (14.84)
Age group (%)			
0-4	0	0	0
5-9	0	0	0
10-14	0	0	0
15-19	2	2	1
20-24	2	2	2
25-29	2	2	2
30-34	3	2	3
35-39	3	3	4
40-44	5	6	5
45-49	9	9	9
50-54	13	12	13
55-59	16	16	15
60-64	20	21	19
65-69	9	11	8
70-74	6	6	6
75-79	9	9	9
80-84	2	2	2
Sex: male (%)	45	44	45
Sex: female (%)	55	56	55
Medical history: general (%)			
Acute respiratory disease	30	30	30
Attention deficit hyperactivity disorder	1	1	1
Long-term liver disease	5	5	5
Long-term obstructive lung disease	7	7	7
Crohn’s disease	1	1	1
Dementia	0	0	0
Depressive disorder	10	10	10
Diabetes mellitus	17	18	17
Gastroesophageal reflux disease	17	17	16
Gastrointestinal hemorrhage	5	5	5
Human immunodeficiency virus infection	2	1	2
Hyperlipidemia	40	40	40
Hypertensive disorder	44	46	43
Lesion of liver	1	1	1
Obesity	8	8	7
Osteoarthritis	19	20	18
Pneumonia	9	11	8
Psoriasis	0	1	0
Renal impairment	9	11	8
Rheumatoid arthritis	3	4	2
Schizophrenia	0	0	0
Ulcerative colitis	1	1	1
Urinary tract infectious disease	10	11	10
Viral hepatitis C	2	2	2
Visual system disorder	32	33	31
Medical history: cardiovascular disease			
Atrial fibrillation	5	6	5
Cerebrovascular disease	3	3	4
Coronary arteriosclerosis	11	12	11
Heart disease	35	38	33
Heart failure	6	6	5
Ischemic heart disease	6	6	7
Peripheral vascular disease	16	17	16
Pulmonary embolism	2	2	2
Venous thrombosis	7	8	7
Medical history: neoplasms (%)			
Hematologic neoplasm	70	75	67
Malignant lymphoma	100	100	100
Malignant neoplasm of anorectum	0	0	0
Malignant neoplastic disease	100	100	100
Malignant tumor of breast	1	1	1
Malignant tumor of colon	0	0	0
Malignant tumor of lung	0	0	0
Malignant tumor of urinary bladder	0	0	0
Primary malignant neoplasm of prostate	1	1	1
Medication use (%)			
Agents acting on the renin-angiotensin system	22	22	21
Antibacterials for systemic use	64	67	61
Antidepressants	16	17	16
Anti-epileptics	10	12	9
Anti-inflammatory and antirheumatic products	21	22	21
Antineoplastic agents	29	39	24
Antipsoriatics	1	1	1
Antithrombotic agents	24	27	22
Beta blocking agents	17	18	17
Calcium channel blockers	11	11	11
Diuretics	19	22	18
Drugs for acid-related disorders	29	33	27
Drugs for obstructive airway diseases	26	27	25
Drugs used in diabetes	10	10	10
Immunosuppressants	6	9	5
Lipid modifying agents	24	24	24
Opioids	44	49	42
Psycholeptics	48	53	45
Characteristic			
Charlson comorbidity index			
Mean	4	4	4
Minimum	2	2	2
25th percentile	2	2	2
Median	3	3	3
75th percentile	5	5	5
Maximum	19	19	17
CHADS2Vasc for stroke prediction			
Mean	2	2	2
Minimum	0	0	0
25th percentile	1	1	1
Median	2	2	2
75th percentile	3	3	3
Maximum	9	9	9
DCSI			
Mean	2	2	2
Minimum	0	0	0
25th percentile	0	0	0
Median	1	1	1
75th percentile	4	4	4
Maximum	13	13	12

Abbreviations: DLBCL, diffuse large B-cell lymphoma; DCSI, Diabetes Complications Severity Index.

Means (SD) or median (IQR) are given for continuous variables; frequencies (percentages) are given for categorical variables: Observation period ⩽1 year after index date.

**Table 4.** Descriptive statistics for DLBCL Cohort 2, observation period ≤1year after index date.

Variable	All subjects (N = 3115)	Outcome (N = 2081)	No outcome (N = 1034)
Age (mean, SD)	56.05 (14.36)	56.88 (14.03)	54.38 (14.89)
Age group (%)			
0-4	0	0	0
5-9	0	0	0
10-14	0	0	0
15-19	2	2	1
20-24	2	2	2
25-29	2	2	2
30-34	3	2	4
35-39	3	2	5
40-44	6	6	6
45-49	9	8	11
50-54	13	12	14
55-59	16	16	16
60-64	19	20	15
65-69	9	10	7
70-74	6	6	5
75-79	9	9	9
80-84	2	2	2
Sex: male (%)	56	56	56
Sex: female (%)	44	44	44
Medical history: general (%)			
Acute respiratory disease	30	30	30
Attention deficit hyperactivity disorder	1	1	1
Long-term liver disease	5	5	6
Long-term obstructive lung disease	6	7	5
Crohn’s disease	1	1	1
Dementia	0	0	0
Depressive disorder	9	10	8
Diabetes mellitus	16	17	14
Gastroesophageal reflux disease	16	17	13
Gastrointestinal hemorrhage	5	5	4
Human immunodeficiency virus infection	1	1	2
Hyperlipidemia	39	39	39
Hypertensive disorder	44	45	42
Lesion of liver	1	1	1
Obesity	7	8	6
Osteoarthritis	18	20	14
Pneumonia	9	10	7
Psoriasis	0	1	0
Renal impairment	9	10	7
Rheumatoid arthritis	3	4	1
Schizophrenia	0	0	0
Ulcerative colitis	1	1	1
Urinary tract infectious disease	11	12	9
Viral hepatitis C	2	1	2
Visual system disorder	32	32	30
Medical history: cardiovascular disease			
Atrial fibrillation	5	6	5
Cerebrovascular disease	3	3	4
Coronary arteriosclerosis	11	11	10
Heart disease	35	37	31
Heart failure	5	6	4
Ischemic heart disease	6	6	7
Peripheral vascular disease	15	16	14
Pulmonary embolism	2	2	2
Venous thrombosis	7	8	7
Medical history: neoplasms (%)			
Hematologic neoplasm	72	75	65
Malignant lymphoma	100	100	100
Malignant neoplasm of anorectum	0	0	0
Malignant neoplastic disease	100	100	100
Malignant tumor of breast	1	1	1
Malignant tumor of colon	0	0	0
Malignant tumor of lung	0	0	0
Malignant tumor of urinary bladder	0	0	0
Primary malignant neoplasm of prostate	1	1	0
Medication use (%)			
Agents acting on the renin-angiotensin system	21	21	21
Antibacterials for systemic use	65	67	61
Antidepressants	16	16	16
Anti-epileptics	10	11	7
Anti-inflammatory and antirheumatic products	22	23	20
Antineoplastic agents	31	36	22
Antipsoriatics	1	1	1
Antithrombotic agents	25	26	23
Beta blocking agents	17	18	16
Calcium channel blockers	11	11	10
Diuretics	20	21	17
Drugs for acid-related disorders	29	31	25
Drugs for obstructive airway diseases	26	27	24
Drugs used in diabetes	9	10	8
Immunosuppressants	7	9	4
Lipid modifying agents	24	24	23
Opioids	45	48	40
Psycholeptics	49	52	42
Characteristic			
Charlson comorbidity index			
Mean	4	4	4
Minimum	2	2	2
25th percentile	2	2	2
Median	3	3	3
75th percentile	5	5	4
Maximum	19	19	17
CHADS2Vasc for stroke prediction			
Mean	2	2	2
Minimum	0	0	0
25th percentile	1	1	1
Median	2	2	1
75th percentile	3	3	3
Maximum	9	9	9
DCSI			
Mean	2	2	2
Minimum	0	0	0
25th percentile	0	0	0
Median	1	1	0
75th percentile	3	4	3
Maximum	13	13	12

Abbreviations: DLBCL, diffuse large B-cell lymphoma; DCSI, Diabetes Complications Severity Index.

Means (SD) or median (IQR) are given for continuous variables; frequencies (percentages) are given for categorical variables: Observation period ⩽3 years after index date.

**Table 5.** Descriptive statistics for DLBCL Cohort 3, observation period ≤1year after index date.

Variable	All subjects (N = 2525)	Outcome (N = 2146)	No outcome (N = 379)
Age (mean, SD)	56.26 (14.06)	56.90 (14.03)	52.65 (14.06)
Age group (%)			
0-4	0	0	0
5-9	0	0	0
10-14	0	0	0
15-19	1	1	1
20-24	2	3	2
25-29	2	2	2
30-34	2	2	4
35-39	3	2	6
40-44	6	6	7
45-49	9	8	13
50-54	13	13	17
55-59	17	16	21
60-64	19	21	9
65-69	10	10	5
70-74	6	6	4
75-79	8	9	7
80-84	2	2	0
Sex: male (%)	56	56	60
Sex: female (%)	44	44	40
Medical history: general (%)			
Acute respiratory disease	30	30	28
Attention deficit hyperactivity disorder	1	1	1
Long-term liver disease	5	5	6
Long-term obstructive lung disease	6	7	4
Crohn’s disease	1	1	1
Dementia	0	0	1
Depressive disorder	9	10	7
Diabetes mellitus	17	18	12
Gastroesophageal reflux disease	17	17	12
Gastrointestinal hemorrhage	5	5	5
Human immunodeficiency virus infection	1	1	3
Hyperlipidemia	39	39	38
Hypertensive disorder	43	45	36
Lesion of liver	1	1	1
Obesity	7	8	4
Osteoarthritis	18	19	12
Pneumonia	10	10	6
Psoriasis	0	1	0
Renal impairment	10	10	8
Rheumatoid arthritis	4	4	1
Schizophrenia	0	0	0
Ulcerative colitis	1	1	1
Urinary tract infectious disease	11	11	11
Viral hepatitis C	2	1	3
Visual system disorder	32	32	28
Medical history: cardiovascular disease			
Atrial fibrillation	5	6	2
Cerebrovascular disease	3	3	2
Coronary arteriosclerosis	11	11	8
Heart disease	35	37	28
Heart failure	5	6	3
Ischemic heart disease	6	6	5
Peripheral vascular disease	15	16	9
Pulmonary embolism	2	2	3
Venous thrombosis	7	7	6
Medical history: neoplasms (%)			
Hematologic neoplasm	73	74	66
Malignant lymphoma	100	100	100
Malignant neoplasm of anorectum	0	0	0
Malignant neoplastic disease	100	100	100
Malignant tumor of breast	1	1	1
Malignant tumor of colon	0	0	0
Malignant tumor of lung	0	0	0
Malignant tumor of urinary bladder	0	0	0
Primary malignant neoplasm of prostate	1	1	1
Medication use (%)			
Agents acting on the renin-angiotensin system	21	21	19
Antibacterials for systemic use	66	67	64
Antidepressants	16	16	18
Anti-epileptics	10	11	6
Anti-inflammatory and antirheumatic products	23	23	21
Antineoplastic agents	34	36	25
Antipsoriatics	1	1	1
Antithrombotic agents	25	26	22
Beta blocking agents	17	18	14
Calcium channel blockers	10	11	8
Diuretics	20	21	15
Drugs for acid-related disorders	30	31	25
Drugs for obstructive airway diseases	27	27	25
Drugs used in diabetes	10	10	7
Immunosuppressants	8	9	4
Lipid modifying agents	24	24	22
Opioids	46	47	39
Psycholeptics	51	51	45
Characteristic			
Charlson comorbidity index			
Mean	4	4	4
Minimum	2	2	2
25th percentile	2	2	2
Median	3	3	3
75th percentile	5	5	4
Maximum	19	19	16
CHADS2Vasc for stroke prediction			
Mean	2	2	1
Minimum	0	0	0
25th percentile	1	1	1
Median	2	2	1
75th percentile	3	3	2
Maximum	9	9	9
DCSI			
Mean	2	2	1
Minimum	0	0	0
25th percentile	0	0	0
Median	1	1	0
75th percentile	3	4	2
Maximum	13	13	9

Abbreviations: DLBCL, diffuse large B-cell lymphoma; DCSI, Diabetes Complications Severity Index.

Means (SD) or median (IQR) are given for continuous variables; frequencies (percentages) are given for categorical variables: Observation period ⩽5 years after index date.

Each cohort was randomly separated into training data and testing data at a ratio of 3:1. Lasso logistic regression (LASSO), Naive Bayes, gradient-boosting machine (GBM), random forest (RF), and neural network models (Supplemental material Table S1) were performed for each cohort. All these prediction models were built using out-of-the-box solutions provided by OHDSI packages. All available clinical and demographic data were included as potential predictors, with no pre-modeling winnowing of potential variables.

To obtain an objective estimation of the algorithms’ performances, baseline prediction models were generated. The first baseline model used a random number generator in the range of 0 to 1 and a threshold. The second and third baseline models were based on a simple attempt to always predict the same outcome (only positive or only negative). All three baseline models produced a useful reference point with which to compare results and will provide information on the benefits of machine-learning algorithms as prediction models in terms of effort versus outcome.

Performance metrics included accuracy, Matthews correlation coefficient, and area under the receiver operating characteristic (ROC) curve (area under the curve [AUC]). Accuracy is a measure of the error rate (ratio of correct predictions to all predictions made). Matthews correlation coefficient is a measure of the quality of binary classifications, where 100% represents a perfect prediction. The ROC curve depicts the true-positive rate (sensitivity) versus the false-positive rate (100%-specificity) at various thresholds, and an AUC of 100% represents a perfect test, and an AUC of 50% indicates non-informative (random) predictions.

## Results

### Descriptive summary

After application of inclusion and exclusion criteria, there were 4501 patients available for Cohort 1 (⩽1 year), 3115 available for Cohort 2 (⩽3 years), and 2525 available for Cohort 3 (⩽5 years). Within these cohorts, there were 1646, 1384, and 2146 patients, respectively, with evidence of progression to a new line of therapy after initial treatment. Although no formal statistical comparison was conducted, descriptive characteristics were similar across all three cohorts (Tables 3 to 5).

### Model comparison

A summary of performance metrics for each predictive model by cohort are shown in Tables 6 to 8. Based on these data, GBM is recommended for predicting progression to later line of therapy after ⩾60 days from the end of a previous therapy or stem cell transplantation in this population of DLBCL patients. When the observation period was ⩽1 year after index date, GBM performed with 67.6% accuracy, a Matthews correlation coefficient of 24.0%, and an AUC of 69.2%. When the observation period was ⩽3 years after index date, GBM performed with 68.0% accuracy, a Matthews correlation coefficient of 21.1%, and an AUC of 72.7%. Accuracy decreased when the observation period was ⩽5 years after index date, as the GBM performed with 84.2% accuracy, a Matthews correlation coefficient of 5.3%, and an AUC of 80.7%.

**Table 6.** Performance metrics across predictive models: observation period ⩽1 year after index date (n = 4501; outcomes = 1646).

Metrics	Lasso logistic regression	Naive Bayes	Gradient-boosting machine	Random forest	Neural network	Random	All positive	All negative
Accuracy, %	66.22	60.89	67.64	66.76	59.47	50.67	63.20	36.80
Matthews correlation coefficient, %	18.98	14.96	23.97	21.20	11.59	−0.44	0.00	0.00
Area under curve, %	68.43	59.96	69.21	68.12	59.04	50.61	50.00	50.00

NOTE: outcome was progression to later line of therapy after ⩾60 days from the end of a previous one or a stem cell transplantation procedure.

**Table 7.** Performance metrics across predictive models: observation period ⩽3 years after index date (n = 3115; outcomes = 2065).

Metrics	Lasso logistic regression	Naive Bayes	Gradient-boosting machine	Random forest	Neural network	Random	All positive	All negative
Accuracy, %	68.29	58.66	68.04	67.68	65.47	49.42	66.37	33.63
Matthews correlation coefficient, %	22.72	20.07	21.13	16.49	20.44	0.07	0.00	0.00
Area under curve, %	71.38	63.96	72.65	68.95	64.78	49.26	50.00	50.00

NOTE: outcome was progression to later line of therapy after ⩾60 days from the end of a previous one or a stem cell transplantation procedure.

**Table 8.** Performance metrics across predictive models: observation period ⩽ 5 years after index date (n = 2525; outcomes = 2126).

Metrics	Lasso logistic regression	Naive Bayes	Gradient-boosting machine	Random forest	Neural network	Random	All positive	All negative
Accuracy, %	84.31	60.54	84.15	84.15	83.52	47.39	13.15	86.84
Matthews correlation coefficient, %	15.09	18.74	5.27	5.27	11.97	−6.53	0.00	0.00
Area under curve, %	77.10	64.79	80.69	76.55	78.99	44.14	50.00	50.00

NOTE: outcome was progression to later line of therapy after ⩾ 60 days from the end of a previous one or a stem cell transplantation procedure.

Detailed model outputs and performance metrics are included as supplementary data (Supplemental material Figure S1 and S2).

## Discussion

This study created a model that considers a large number of independent variables to predict health outcomes after treatment or autologous stem cell transplantation in patients with DLBCL. Predictive models based on GBM and observation periods ⩽1 and ⩽3 years after index date were the best-performing algorithms. The predictive model was generated efficiently using a large number of independent variables readily available in standard insurance claims or electronic health record data systems. Within this study, outcomes assessment was simplified as binary (progression to new treatment vs non-progression) within fixed time windows, but future enhancements could also include prediction of variation in time-to-event outcomes. Validation in a 25% test hold-out sample was performed to reduce risk of over-fitting and to calculate ROC curves and Matthews correlation coefficients. As a next step, further validation could be conducted in independent data sets, thereby further ensuring robustness of model accuracy. Replication in clinically richer data sources, such as oncology-specific electronic health record databases or clinical trial data sets, could further provide opportunity to enhance model accuracy.

Established uses of prognostic modeling include point-of-care treatment decision-making and the identification of patients who warrant closer follow-up. For instance, a provider may select an alternative treatment for a patient identified as having a high likelihood of treatment response for a given therapy,^23,24^ as by Porcher et al,^25^ for additional radiotherapy in soft-tissue sarcoma. Predictive models such as the one developed here may also facilitate more efficient clinical development of investigational drugs in DLBCL. It could be utilized for the enrichment of the patient population recruited into clinical trials in DLBCL where the goal is to focus on patients with a lower likelihood of response to standard of care. In a hypothetical clinical trial of an investigation drug versus standard of care in DLBCL, the estimated necessary sample size to demonstrate therapeutic effect within 1 year of treatment when assuming a treatment arm response rate of 40% and a standard of care arm response rate of 20%, is 109 patients per arm (standard two-sample test for proportions; assuming a beta of 0.9 and alpha of 0.05). Applying GBM to recruit patients with a low likelihood of treatment response to standard of care at a sensitivity of 0.60 and specificity of 0.68 reduces the response rate to 12% in the standard of care arm. Assuming that the treatment arm response rate is unchanged, the expected magnitude of effect between arms is increased by 11 percentage points, reducing the required sample size to 50 patients per arm. Realistically, the treatment arm response rate would also be expected to decrease. To model this decrease, all patients who respond to standard of care are also expected to respond to the new treatment. In addition, a fraction of patients who do not respond to standard of care will not respond to the new treatment, independent of patients’ baseline covariates. Even assuming treatment arm response at 34%, there is a net decrease in sample size to 75 patients per arm. When considering all scenarios, applying a predictive model for response rate to standard of care could reduce the sample size of this hypothetical clinical trial in DLBCL by 33 to 68 patients, which would readily translate into reduced costs and time needed to accrue trial patients. This is particularly impactful for oncology trials where recruitment has become increasingly difficult, and costs per patient have ranged from US$68 500 to US$125 000 and continue to increase.^26–28^

The predictive model also provides the opportunity to implement a more systematic approach to the treatment of DLBCL patients. The model may inform clinical decision-making, allowing the identification of patients most likely to respond to a specific drug or drug combination,^9^ support more accurate diagnoses, avoid unnecessary treatments and associated adverse effects, and decrease the burden of DLBCL disease. Notably, health care resource utilization and costs are significantly higher in patients with DLBCL who progress after first-line therapy compared with those without relapse or refractory disease. Evidence from the MarketScan database identified chemotherapy and autologous stem cell transplantation in second-line therapy as major drivers of DLBCL health care costs.^8^ An analysis of Medicare claims data in adults >65 years revealed that patients with DLBCL who relapsed after first-line therapy had significantly higher rates of inpatient hospital admissions (60.7% vs 41.1%), emergency department visits (51.7% vs 43.0%), and use of skilled nursing facility (19.3% vs 12.5%), home health agency (35.5% vs 23.3%), and hospice services (19.9% vs 6.3%), resulting in higher total all-cause health care costs of US$6566 per relapsed patient per month, compared with US$1951 in non-relapsed patients.^29^ Taken together, these data suggest that a predictive model of relapse or the presence of refractory disease in patients with DLBCL has the potential to increase the efficiency of DLBCL health care delivery, lessen the impact of DLBCL on health care systems by lowering the overall cost of DLBCL health care, and reduce DLBCL patient burden by decreasing the need for health agency and hospice care.

An additional application of such modeling approaches can be to identify new variables or factors for predicting outcomes. The exploration of variables or patterns of variables identified as top predictors across multiple modeling approaches could be considered as a way to generate hypotheses for new predictive factors for a given outcome. Any assertions of causality, however, would require employing causal inference methodologies,^30^ which are outside the scope of this study.

The framework used to develop the predictive model described in this study can overcome data sparseness, may help to generate new hypotheses for predictors of outcomes, and can be readily implemented to efficiently develop a predictive model for measurable outcomes; however, the framework is associated with several limitations. First, censored patients cannot be included, so any individual who is not observed for the complete follow-up period or experiences an outcome during follow-up is excluded, which may introduce bias in the study population. Second, not all medical events are recorded in observational data sets and some information can be recorded incorrectly, resulting in a noisy data set with potential outcome misclassification. Third, the resultant predictive model is only applicable to the population of patients represented by the data used to train the model; therefore, generalization may be limited. Finally, a limitation of any model used for clinical trial enrollment is the need to have access to all variables at the time of screening.

## Conclusions

This study developed a model that considers a large number of independent variables to predict health outcomes in patients with DLBCL. The model has potential application for enriching the patient population recruited into clinical trials in DLBCL, where the goal is to focus on patients with lower likelihood of response to standard of care, improving efficiencies in the delivery of health care to patients with DLBCL and reducing health care costs.

## Supplemental Material

Supplemental_material – Supplemental material for Predicting Outcomes in Patients With Diffuse Large B-Cell Lymphoma Treated With Standard of Care Click here for additional data file. Supplemental material, Supplemental_material for Predicting Outcomes in Patients With Diffuse Large B-Cell Lymphoma Treated With Standard of Care by Aaron Galaznik, Christian Reich, Greg Klebanov, Yuriy Khoma, Eldar Allakhverdiiev, Greg Hather and Yaping Shou in Cancer Informatics
