# Supplementary material for: Predicting Outcomes in Patients With Diffuse Large B-Cell Lymphoma Treated With Standard of Care
Source: Cancer Inform. 2019 Mar 15;18:1176935119835538. doi: 10.1177/1176935119835538 (PMC6421613; doi:10.1177/1176935119835538)
Supplement: Supplemental_material – Supplemental material for Predicting Outcomes in Patients With Diffuse Large B-Cell Lymphoma Treated With Standard of Care [file Supplemental_material.pdf]

SUPPLEMENTARY INFORMATION

Table S1 Predictive models: Overview

Predictive model	Description
LASSO (Least Absolute Shrinkage and Selection Operator)	Models the dependent variable while automatically selecting significant variables by shrinking the coefficients of unimportant predictors to zero.
Naive Bayes	Assumes the value of a particular feature is independent of the value of any other feature
Gradient boosting machine	Captures complex non-linear function dependencies builds an ensemble of trees one-by-one, then the predictions of the individual trees are summed
Random forest	Operates by constructing multiple decision trees; the sum of the predictions made from the decision trees determines the overall prediction of the forest.
Neural network model	Organized in a series of layers, where the input vector enters at the left side of the network, which is then projected to a "hidden layer." Each unit in the hidden layer is a weighted sum of the values in the first layer. This layer then projects to an output layer

Figure S1 Gradient Boosting Machine: Performance metrics, observation period ≤1 year post-index date

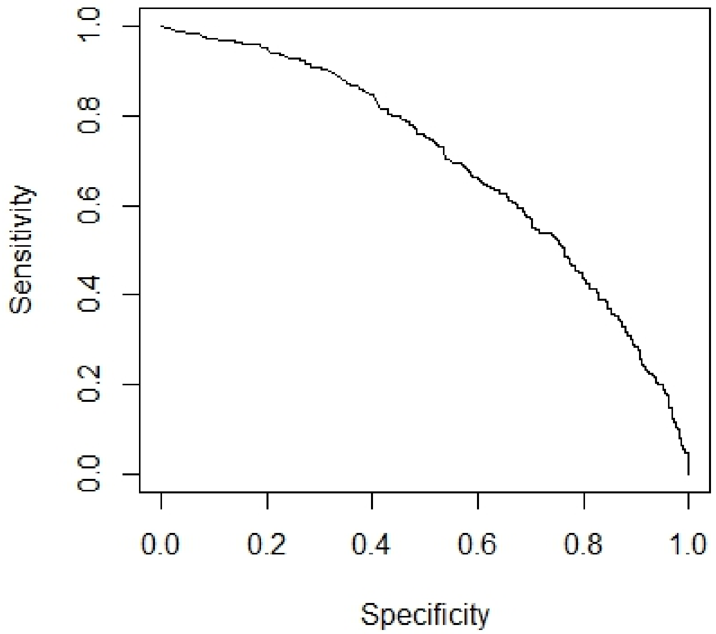

	observed	
predicted	0	1
0	483	165
1	232	245

sensitivity: 0.597561
specificity: 0.6755245

Figure S2 Gradient Boosting Machine: Performance metrics, observation period ≤3 years post-index date

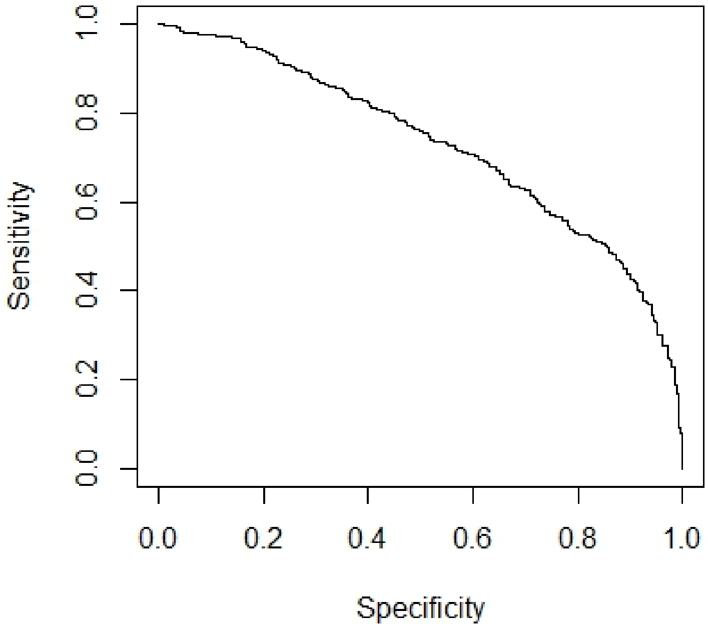

	observed	
predicted	0	1
0	226	262
1	37	254
